# Penetrating Aortic Injury due to Broken Ribs and Preventive Measures

**DOI:** 10.1055/s-0042-1757946

**Published:** 2022-12-20

**Authors:** Youichi Yanagawa, Hiroki Nagasawa, Kouhei Ishikawa, Shunki Hirayama, Akira Itoi, Atsuhiko Mogami

**Affiliations:** 1 Department of Acute Critical Care Medicine, Juntendo University, Shizuoka Hospital, Shizuoka, Japan; 2 Department of General Thoracic Surgery, Juntendo University, Shizuoka Hospital, Shizuoka, Japan; 3 Department of Orthopedics, Juntendo University, Shizuoka Hospital, Shizuoka, Japan

**Keywords:** fractured rib, penetrating aortic injury, operation

## Abstract

We herein report two cases of patients that underwent prophylactic operations to prevent aortic injuries in association with fractured ribs. Penetrating aortic injuries induced by fractured ribs remain fatal. Prophylactic operations appear effective. However, the indication for such operations should be clarified further in the future.

## Introduction

Thoracic aortic injury caused by rib fractures is rare. In such cases, emergency surgery is usually required due to unstable circulation. 1 The location of left-sided posterior rib fractures from flail chest is a risk factor for penetrating aortic injury. 2 Some patients show delayed aortic penetrating injury due to moving fractured ribs. Prophylactic operations to prevent such injuries have been reported. 3 As no reviews have focused on penetrating aortic injury due to fractured ribs and preventive measures, we herein report our cases and review the relevant literature. The protocol of this study was approved by our institutional review board, and the examinations were conducted according to the standards of good clinical practice and the Declaration of Helsinki.

## Case Presentations

### Case 1

**Fig. 1:**
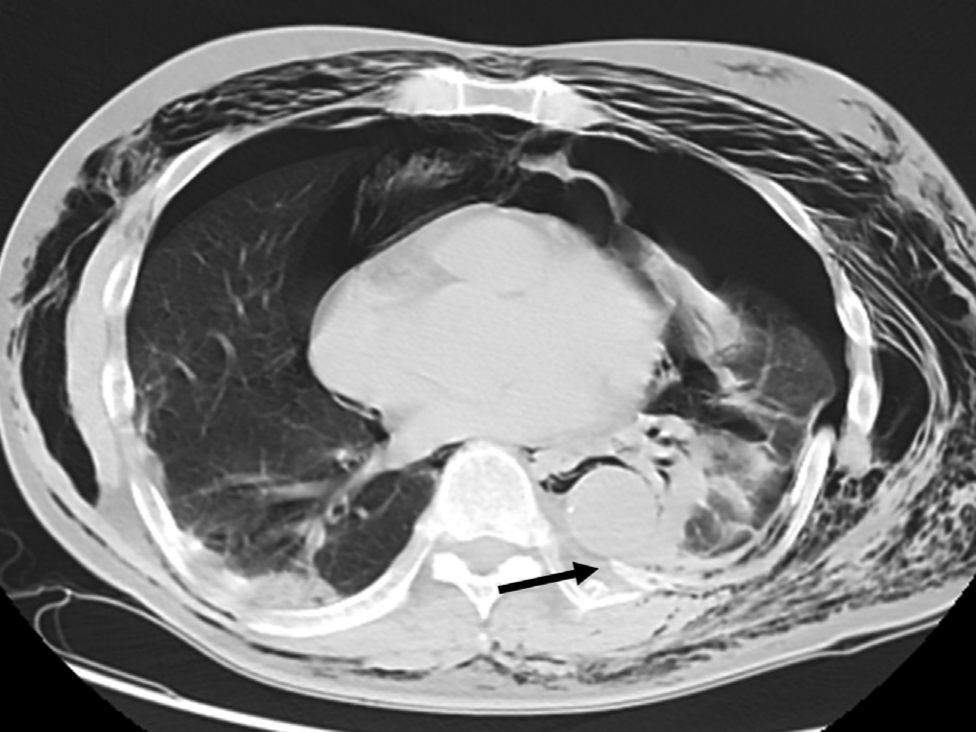
Thoracic computed tomography (CT) on arrival in case 1. CT shows the spiculated end of the ninth fractured rib adjacent to the descending aorta (arrow).

A 65-year-old man who sometimes lost his memory after drinking alcohol noticed left chest and neck pain after the ingestion of a massive amount of alcohol. His symptoms did not improve after 2 days, and he could not walk by himself. He, therefore, called an ambulance. He was transported to our hospital by ground and air ambulance due to a severe hypoxic state. On arrival, he had a clear consciousness. Physical examination revealed the following: blood pressure, 170/118 mm Hg; heart rate, 122 beats per minute; percutaneous oxygen saturation, 97% on 10 L per minute of oxygen by mask; and body temperature, 37.7°C. He had subcutaneous hemorrhage in the left chest with flail chest and severe subcutaneous emphysema at the upper trunk. Whole body computed tomography (CT) with contrast revealed multiple left rib fractures (II–XII), bilateral hemopneumothorax, left lumbar transverse process fractures (I–III), left clavicular fracture, and right renal cystic injury. In addition, the ninth fractured rib was adjacent to the descending aorta ( Fig. 1 ). He underwent bilateral thoracostomy. He was admitted to the intensive care unit (ICU) under the prohibition of the left decubitus position. On day 2, his hypoxia deteriorated due to atelectasis and he underwent tracheal intubation with mechanical ventilation for internal fixation. He underwent tracheostomy on hospital day 3, removal of the fifth fractured rib on the hospital day 5, and internal fixation for left clavicular fracture on the 8th hospital day. On the 12th day, mechanical ventilation was withdrawn and the tracheal tube was removed on the 14th hospital day. After rehabilitation, he was discharged on foot on hospital day 23.

### Case 2

**Fig. 2:**
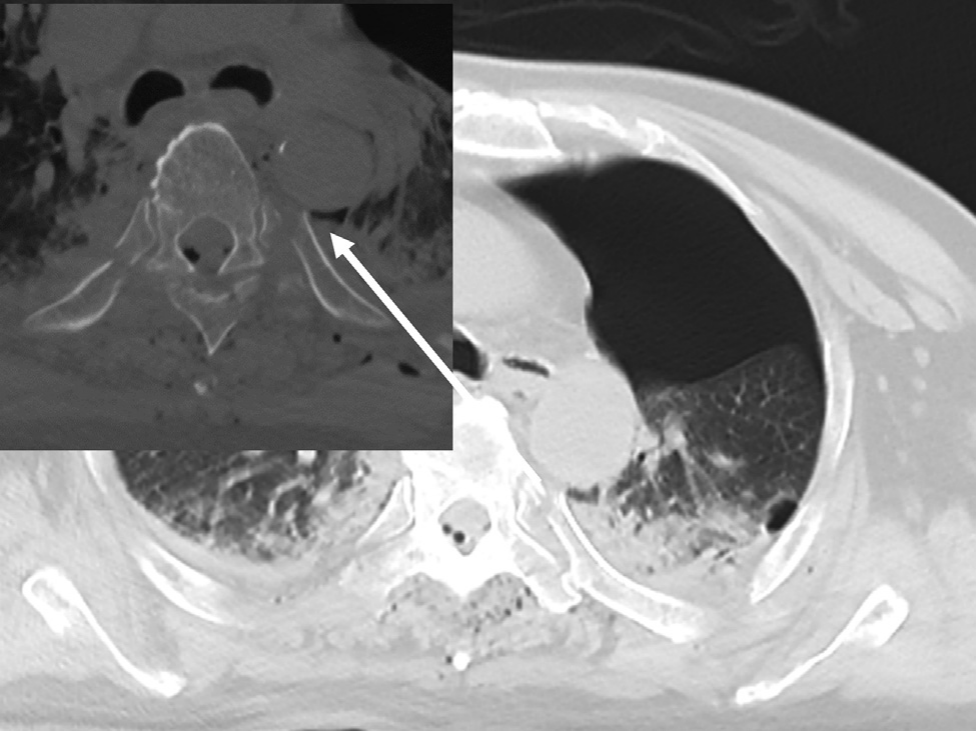
Thoracic computed tomography (CT) on arrival in case 2. CT shows the spiculated end of the fifth fractured rib adjacent to the descending aorta (arrow).

A 76-year-old man with hypertension was knocked more than 3 m by a car moving at the speed of 30 km per hour. He was transported to our hospital by ground and air ambulance. On arrival, he had clear consciousness. Physical examination revealed the following: blood pressure, 127/85 mm Hg; heart rate, 114 beats per minute; percutaneous oxygen saturation, 92% on 10 L per minute of oxygen by mask; and body temperature, 35.0°C. He had left chest pain and tenderness with flail chest. Whole body CT with contrast revealed multiple left rib fractures (I–X), multiple right rib fractures (I, V–VII), bilateral hemopneumothorax, unstable thoracic spinal fractures (IV and V), traumatic subarachnoid hemorrhage, and left foot fracture. In addition, the fifth fractured rib was adjacent to the descending aorta ( Fig. 2 ). He underwent left thoracostomy, tracheal intubation, and mechanical ventilation for internal fixation. He was admitted to the ICU under the prohibition of left decubitus position. He underwent posterior internal fixation for the thoracic spine with screws and rods and fixation of the fifth fractured rib with wires on hospital day 7, tracheostomy on hospital day 9, and internal fixation for foot fracture on hospital day 14. Removal of the tracheal tube failed due to difficult excretion of sputum, and he was transferred to another hospital for rehabilitation.

## Discussion

There are only seven previous reports on prophylactic operations to prevent the aortic injuries induced by fractured ribs. 1 3 4 5 6 7 8 We summarized these cases, including the present two cases in Table 1 . As a result, prophylactic surgery appears to be effective. In addition, evaluation using axial CT images is necessary to evaluate the relationship between the edge of the fractured ribs and the descending aorta, at least for patients with flail chest. The remaining clinical questions concern the indications for prophylactic operations, the operative method, and alternate conservative treatment, such as internal fixation for flail chest using mechanical ventilation with positive end-expiratory pressure. Concerning the indications for prophylactic operations, fractured ribs in contact with the descending aorta are considered to be an absolute indication for operation. The minimal safe distance between the edge of the fractured rib and the descending aorta has not yet been investigated. In addition, internal fixation for flail chest using mechanical ventilation with positive end-expiratory pressure may be useful for preventing penetrating aortic injuries induced by fractured ribs. Our department pursues such management for patients when the distance from the edge of the fractured ribs to the descending aorta exceeds 1 to 3 cm. At the present, the decision to operate depends on the consent of both the patient and surgeon. In such cases, the development of a scale would be helpful, including such factors as radiological or anatomical findings that could be considered “high risk,” thereby indicating the need to perform prophylactic procedures. However, developing such a scale is currently impossible due to the fact that there are just too few patients with data that can be fully evaluated. Further accumulation of case reports on penetrating aortic injuries induced by fractured ribs is necessary to resolve the remaining clinical questions.

**Table 1:** Previous reports on prophylactic operations to prevent the aortic injuries induced by fractured ribs

No.	Author	Year	Age (y)	Sex	Mechanism of injury	Penetration	Ribs	Flail chest	Chest tube drainage	Mechanical ventilation	Complication	Treatment	Outcome
1	Zhao et al ^1^	2021	54	F	Falling object	No	5,6,7,8	No	No	No	Lung, spine	Internal fixation	Survival
2	Bartscherer et al ^3^	2019	21	F	Train accident	No	5	Yes	Yes	Yes	Lung, spleen, extremity	Plate	Survival
3	Uemura et al ^4^	2016	19	F	Traffic accident	No	9,10	No	Yes	Yes	Lung, scapula, spine, liver, pelvis	Resection of fractured rib	Survival
4	Funaki et al ^5^	2014	66	F	Traffic accident	No	8	No	Yes	No	Lung	Video-assisted thoracoscopic resection of fractured ribs	Survival
5	Kobayashi et al ^6^	2012	81	M	Traffic accident	No	7	Yes	Yes	Yes	Lung	Resection of fractured rib	Survival
6	Carter et al ^7^	2011	43	F	–	No	5	–	No	No	Lung	Resection of rib	Surivival
7	Sata et al ^8^	2007	50	M	Building accident	No	9	Yes	Yes	Yes	Lung, spleen	Repair of flail chest	Survival
8	Present		65	M	Fall	No	9	Yes	Yes	Yes	Lung, clavicular, kidney	Resection of fractured rib	Survival
9	Present		76	M	Traffic accident	No	10	Yes	Yes	Yes	Lung, clavicular, kidney	Resection of fractured rib	Survival

Abbreviations: F, female; M, male.

Penetrating aortic injuries induced by fractured ribs remain potentially fatal. Prophylactic operations appear effective; however, the concrete indications of such operations remain to be clarified.
